# Effects of *Ferulago angulata* Extract on Serum Lipids and Lipid Peroxidation

**DOI:** 10.1155/2014/680856

**Published:** 2014-02-24

**Authors:** Mahmoud Rafieian-kopaei, Najmeh Shahinfard, Hojjat Rouhi-Boroujeni, Mojgan Gharipour, Pariya Darvishzadeh-Boroujeni

**Affiliations:** ^1^Medical Plants Research Center, Shahrekord University of Medical Sciences, Shahrekord, Iran; ^2^Isfahan Cardiovascular Research Center, Isfahan University of Medical Sciences, Isfahan, Iran; ^3^Lund University, Lund, Sweden

## Abstract

*Background*. Nowadays, herbs they are considered to be the main source of effective drugs for lowering serum lipids and lipid peroxidation. The present experimental animal study aimed to assess the impact of *Ferulago angulata* on serum lipid profiles, and on levels of lipid peroxidation. *Methods*. Fifty male Wistar rats, weighing 250–300 g, were randomly divided into five equal groups (ten rats in each). The rat groups received different diets as follows: Group I: fat-rich diet; Group II: fat-rich diet plus hydroalcoholic extracts of *Ferulago angulata* at a dose of 400 mg/kg; Group III: fat-rich diet plus hydroalcoholic extracts of *Ferulago angulata* at a dose of 600 mg/kg; Group IV: fat-rich diet plus atorvastatin; Group V: common stock diet. The levels of serum glucose and lipids and the atherogenic index were measured. In addition, malondialdehyde (MDA), thiol oxidation, carbonyl concentrations, C-reactive proteins, and antioxidant capacity were evaluated in each group of rats. *Results*. Interestingly, by adding a hydroalcoholic extract of *Ferulago angulata* to the high-fat diet, the levels of total cholesterol and low-density lipoproteins (LDL) in the high-fat diet rats were both significantly reduced. This result was considerably greater compared to when atorvastatin was added as an antilipid drug. The beneficial effects of the *Ferulago angulata* extract on lowering the level of triglycerides was observed only when a high dosage of this plant extraction was added to a high fat diet. Furthermore, the level of malondialdehyde, was significantly affected by the use of the plant extract in a high-fat diet, compared with a normal regimen or high-fat diet alone. *Conclusion*. Administration of a hydroalcoholic extract of *Ferulago angulata* can reduce serum levels of total cholesterol, triglycerides, and LDL. It can also inhibit lipid peroxidation.

## 1. Introduction

Cardiovascular diseases are one of the main causes of death or life-threatening morbidities throughout the world. Hyperlipidemia is a major risk factor for these disorders and it is closely associated with the appearance and progression of coronary atherosclerosis [1]. Various antilipid medications are now used to lower serum lipids in patients with suspected cardiac ischemic disease; however, due to a range of associated serious adverse events such as liver disease, severe muscular disorders, fetal complications, drug interactions, and also the impossibility of using these drugs for long periods of time, the administration of these drugs has now been limited. Therefore, the use of herbal drugs, which have frequently shown promising effects in the treatment of various complications, such as diabetes mellitus [2], cancer [3, 4], Alzheimer's [5, 6], and gastrointestinal complications [7, 8], is preferable.

Herbs are now considered to be a main source for preparing the most effective drugs for lowering serum lipids. More than 200 types of herbal drugs have been identified to have antilipid effects; however, these effects have not been confirmed in a notable number of them.

*Ferulago angulate* (locally called Chavil) is widespread in the high altitudes of several Asian countries such as Turkey, Iraq, and Iran [9]. Data in the existing literature state that oil originating from this plant has different antimicrobial and antioxidant properties, which can be beneficial in those who are susceptible to ischemic cardiac events [10, 11]. In addition, during the last decade, several lines of evidence have suggested that low-density lipoprotein (LDL) oxidation is a key mechanism for rendering lipoproteins atherogenic [11]. According to the demonstrated role of *Ferulago angulata* as an antioxidant plant [11], and due to the association of oxidized lipids with the progression of atherosclerosis, the role of *Ferulago angulata* in lowering serum lipids and thus preventing the formation of atherosclerosis is now hypothesized. Hence, the present experimental animal study aimed to assess the effects *of Ferulago angulata* on serum lipid profiles and on the levels of lipid peroxidation.

## 2. Methods

### 2.1. Animal Selection

In this experimental study, 50 Wistar male rats weighing 250–300 g were divided into five equal groups as follows: Group I received a fat-rich diet with a Presintra-M regimen (containing serum itralipid) (a type of rat diet); Group II received a fat-rich diet with Presintra-Mregimen, plus hydroalcoholic extracts of *Ferulago angulata *at a dose of 40 mg/kg; Group III received a fat-rich diet with a Presintra-M regimen, plus hydroalcoholic extracts of *Ferulago angulata *at a dose of 600 mg/kg; Group IV: received a fat-rich diet with a Presintra-M regimen plus atorvastatin (10 mg/kg); Group V: received only a routine rat diet. The housing conditions, including room temperature and light and dark cycles, were identical for all groups of rats throughout the study. All rats in the study survived for at least 40 days and were fed during this period. In order to prepare the high-fat diet, the Presintra-M regimen was prepared from egg yolk cholesterol (1 g cholesterol from 100 g egg yolk) and serum itralipid reaching 2% cholesterol combined with triglycerides and choline in a palm oil-based emulsion. Finally, an appropriate dose of this regimen to induce hypercholesterolemia in animals (25 mg/kg/day) was administered [12] orally by gavage.

### 2.2. Preparation of Plant Extract

A herbarium specimen of *Ferulago angulata *was prepared and deposited in the Medical Plants Research Center Herbarium of Shahrekord University of Medical Sciences, Iran.

Initially, the plant was ground into a powder and passed through a suitable sieve. Then, through the following percolation method, a hydroalcoholic extract of the plant was prepared. This process continued for 72 hours; when the extraction was completed, it was followed by evaporation of the alcohol using a rotary evaporator [13]. Finally, two different concentrations of a hydroalcoholic extract of *Ferulago angulate* (400 mg/cc and 600 mg/cc) were prepared.

### 2.3. Study Measurements

All of the blood samples were centrifuged at 3500 g for 20 min, after the blood had been collected directly from the rat's heart. Blood glucose and serum lipids were measured using commercially available kits from Pars Azmoon (Tehran, Iran). Serum samples were analyzed with a BT 3000 Plus biochemical analyzer (Biotecnica, Italy). To investigate the possible antioxidant effects of the plant, the amount of malondialdehyde (MDA) (as a marker for assessing lipid peroxidation) was measured using a reverse phase high-pressure liquid chromatography (HPLC) method, after derivatization with 2,4-dinitrophenylhydrazine. Thiol oxidation and carbonyl concentrations were measured by spectrophotometry. C-reactive protein (CRP) as an acute phase protein was determined by using commercial animal study kits (Pars-Azmoon, Iran). Antioxidant capacity was evaluated by the DPPH method [14]. The atherogenic index of plasma (AIP = Log(TG/HDL_C)) is defined as the zone of atherogenic risk.

### 2.4. Statistical Analysis

In order to compare the measured biomarkers among the animal groups, a one-way ANOVA test or a Kruskal-Wallis test was used. In statistical analysis of the data, *P* values of 0.05 or less were considered statistically significant. All the statistical analyses were performed using SPSS (version 19) for Windows (SPSS Inc., Chicago, IL, USA).

## 3. Results

As demonstrated in Table 1, by using a high-fat diet, either with a plant extract regimen or without it, blood sugar levels were increased significantly and thus adding plant extract to a high cholesterol regimen could not effectively reduce blood glucose levels. However, total cholesterol and also the level of LDL were both significantly reduced by adding plant extract to a high-fat diet, which was even more effective than when atorvastatin was added as an antilipid drug. This beneficial effect of plant extract was not revealed on HDL concentrations and was observed in triglycerides only with high dosages of this plant extraction. Furthermore, the level of MDA as an oxidant agent could be strongly affected by the use of the plant extract in a high-fat diet compared with a normal regimen or high-fat diet alone.

Table 2 summarizes the results and compares the biochemical factors in all of the study groups. The two groups which received a normal diet and a high-fat diet plus atorvastatin had approximately the same levels of ferritin, thiol, MDA, and carbonyl, whereas the group supplied with a high-fat diet had elevated levels of all of the above factors. In contrast, the groups which received a high-fat diet plus plant diet (400 or 600 mg) had lower levels of MDA, CRP, and ferritin, and higher levels of antioxidant capacity and thiol activity, compared to the other groups. Moreover, the rats fed with a high-fat diet plus the plant diet (600 mg) were shown to have significantly higher levels of antioxidant capacity, in comparison with the rats which received the high-fat diet plus lower doses of the plant diet (400 mg).

## 4. Discussion

Our study demonstrated that using *Ferulago angulata* is associated with lower MDA and CRP levels and higher antioxidant capacity and thiol activity in the study groups. Using higher doses of *Ferulago angulata* had a stronger effect on the hyperlipidemic rats than on the other groups. Epidemiological studies strongly suggest that the long-term consumption of diets rich in plant polyphenols offer protection against the development of cancers, cardiovascular diseases, diabetes, osteoporosis, and neurodegenerative diseases [15]. Along with a few previously published studies on the importance of the therapeutic properties of *Ferulago angulata* in the medicinal field, first we attempted to confirm the antioxidant effect of this plant extract and then we examined its antilipid properties, leading to a demonstration of its protective effects against cardiovascular risks.

According to previous results, this plant has 25 various components with different antibacterial, anti-inflammatory, and antioxidant effects [16]. Our study demonstrated a significant effect of *Ferulago angulata* on plasma MDA levels, which is a major oxidant marker in animal models that were fed with a high-fat regimen. This antioxidant effect was evident in those groups fed with higher doses of the plant extract. In a study by Amirghofran et al. [17] an extract of this plant, at a concentration of 50 *μ*g/mL, demonstrated a significant decrease in nitric oxide production after a 24-hour treatment. This inhibitory effect was also observed after 48 hours. Vast experimental studies have proven the antioxidant properties of *Ferulago angulata*. In this regard, peroxide and thiobarbituric indexes of the samples were determined and compared with blank samples (without any antioxidants and with tertiary butyl hydroquinone (TBHQ)). The results indicated that the minimum concentration of extract for conserving the vegetable oil is about 0.02% under excremental conditions [18]. As a result of convincing evidences for the contribution of oxidative damage to the pathology of atherosclerosis and vascular defects, the antioxidant protective effects of this plant make an important contribution to its cardiovascular protective effects.

In addition to the antioxidant properties of *Ferulago angulata*, based on the key role of lipid peroxidation in the progression of atherosclerosis, for the first time, we hypothesized that the antioxidant effect of this plant might inhibit lipid peroxidation and thus the level of oxidized lipids might be reduced by administrating an extract of this plant. To demonstrate this hypothesis, we measured the serum levels of lipid profiles, as well as biochemical biomarkers of oxidative capacity, in animals fed with a high-fat regimen in combination with different doses of *Ferulago angulata *extract. The results indicated that the level of total cholesterol and also LDL were both significantly reduced by adding plant extract to a high-fat regimen, and this lipid lowering effect could be comparable with the effects of atorvastatin, which is a strong antilipid drug. We believe that the effects of *Ferulago angulata* might not originate from its direct effect on lipid metabolism; however, it could be mediated by its influence on lowering oxidized lipid products. However, this is the first observation on the antilipid effects of *Ferulago angulata* and thus it needs to be extensively evaluated in further studies.

In conclusion, an extract of *Ferulago angulata *can effectively increase the activity of thiol groups, reduce plasma levels of MDA as an oxidant agent, and increase antioxidant capacity and it can also decrease serum lipids. Therefore, using an extract of this plant might have a strong cardioprotective effect. However, in order to minimize its side effects, the optimal dosages and the time of administration of this plant extract need to be assessed in further trials.

## Figures and Tables

**Table 1:** Comparison of biochemical markers between the experimental study groups.

	Glucose (mg/dL)	Total cholesterol (mg/dL)	Triglyceride (mg/dL)	LDL (mg/dL)	HDL (mg/dL)	VLDL (*μ*mol/L)	Atherogenic index (mg/dL)
Normal diet	130.66 ± 48.52	55.22 ± 14.06	89.77 ± 27.01	22.48 ± 4.68	27.53 ± 9.27	17.95 ± 1.22	0.153 ± 0.062
High-cholesterol diet	187.55 ± 12.19	119.00 ± 7.19	178.55 ± 15.32	52.38 ± 5.08	42.27 ± 1.19	35.71 ± 1.56	0.266 ± 0.041
Plant diet (400 mg)	177.67 ± 38.15	81.40 ± 13.28	145.61 ± 12.97	34.71 ± 3.21	41.46 ± 6.02	29.12 ± 7.32	0.175 ± 0.012
Plant diet (600 mg)	166.87 ± 30.21	73.21 ± 10.93	102.33 ± 7.42	22.49 ± 4.05	42.65 ± 5.59	20.46 ± 0.21	0.020 ± 0.003
Atorvastatin	214.40 ± 44.80	76.20 ± 6.45	132.21 ± 14.14	32.58 ± 7.23	42.60 ± 6.26	27.38 ± 1.12	0.023 ± 0.054

HC: high cholesterol.

AIP < 0,11: low risk.

AIP (0.11–0.21): intermediate risk.

AIP >0.22: increased risk.

**Table 2:** Comparison of biochemical activity between the experimental study groups.

	Normal diet	High-cholesterol diet	Plant diet (600 mg)	Plant diet (400 mg)	Atorvastatin	*P* value
Ferritin	2.7 ± 0.04	9.1 ± 1.43	2.9 ± 0.07	3.6 ± 0.15	2.7 ± 0.08	<0.05
Thiol plasma	201.3 ± 0.56	173.6 ± 0.38	217.8 ± 9.43	209.5 ± 8.01	207.05 ± 0.7	<0.05
Malondialdehyde	5.74321 ± 0.32	10.03581 ± 0.70	4.30072 ± 0.66	5.64488 ± 0.85	5.11525 ± 0.73	<0.05
Carbonyl plasma	0.72 ± 0.05	0.89 ± 0.08	0.71 ± 0.02	0.75 ± 0.04	0.72 ± 0.08	<0.05
CRP	3.14 ± 0.22	3.98 ± 0.3	3.00 ± 0.2	3.40 ± 0.078	3.00 ± 0.23	<0.05
Antioxidant capacity	282.2 ± 4.23	432.1 ± 1.72	462.2 ± 3.86	413.3 ± 5.54	399.7 ± 0.48	<0.05

CRP: C-reactive protein.
